# From Underground to Mainstream: Establishing a Medical Lexicon for Psychedelic Therapy

**DOI:** 10.3389/fpsyt.2022.870507

**Published:** 2022-06-17

**Authors:** Andrew Beswerchij, Dominic Sisti

**Affiliations:** Department of Medical Ethics and Health Policy, Perelman School of Medicine, University of Pennsylvania, Philadelphia, PA, United States

**Keywords:** psychedelic psychiatry, psychedelics, language, stigma, treatment process, narrative medicine, hallucinogens, psychopharmacological treatments

## Abstract

We argue that non-stigmatizing and precise terminology grounded in the medical model will advance both the science and public acceptance of psychedelics. Researchers and clinicians should take care to distinguish between medical, recreational, and spiritual uses to set clear boundaries and expectations for patients. Ethically fraught or stigmatizing terms should be replaced with terminology that is medically and scientifically descriptive and accurate. A medicalized linguistic framework around psychedelics will potentially yield benefits and mitigate risks. Replacing colloquial names with scientific names for medicines and therapies may help correct misconceptions about psychedelics commonly held by both professionals and the public. A harmonized medical lexicon will also provide a common language for important instances of communication—such as the informed consent process—between professionals and participants. Our recommendations draw upon communications research in addiction medicine and aim to encourage the development, acceptance, and implementation of non-stigmatizing terminology in psychedelic research and treatment.

## Introduction

Research in psychedelic therapy is rapidly advancing with the first FDA-approved medicines expected to arrive within the next 2–4 years. As an emerging clinical specialty, psychedelic therapy has attracted newly interested practitioners, researchers, educators, and investors. The future of the psychedelic renaissance is becoming clear, as new psychedelic treatment clinics open across the US. At the moment, these clinics only offer ketamine therapy for the treatment of major depressive disorder (MDD), post-traumatic stress disorder (PTSD), and other conditions. With the eventual approval of MDMA and psilocybin, market forecasts predict rapid growth across the psychedelic-based mental healthcare sector, from $2 billion in 2019 to $7–10 billion in 2027 (1).

Important aspects of the previous psychedelic era continue to undermine progress in studying the therapeutic potential of these molecules. Most obviously, the social, juridical, and regulatory structures established during the War on Drugs have created barriers and continue to impede psychedelics research (2). In the US, psychedelics remain Schedule I substances—a category reserved for drugs that are thought to offer no medical benefit and are considered highly addictive. This policy also reflects the general public's misunderstanding and attitudes about these substances.

Psychedelic medicines intended to treat mental disorders are doubly stigmatized. In addition to the negative misconceptions rooted in the 1960 s counterculture and drug policies, mental illness is in general highly stigmatized (3–6). Stigma attaches to individuals through, among other things, the words we use to label and describe them and their associated activities or characteristics (7). The medical interventions related to mental illness become part of the stigmatizing dynamic, with particular words reinforcing negative assumptions about the use of psychotropic medication, electroconvulsive therapies, inpatient care, etc.

The anesthetic ketamine has dissociative and psychoactive properties and has been used off-label for various psychiatric conditions since the 1970 s. A stereoisomer of ketamine has recently been approved by the FDA for clinical use in depression (8). Research organizations are navigating the conventional regulatory pathway toward FDA approval to bring these medicines to market. This is sometimes referred to as the “medical model” that is in contrast to approaches aimed at legalization for recreational, spiritual, or off-label medical uses. To avoid confusion between these other uses and FDA-approved ones, and to likewise expand the public's access to these medicines, a scientifically informed lexicon for psychedelic research and treatment appears to be necessary.

In this paper, we argue that researchers, clinicians, and patient-interfacing companies should carefully develop and adopt a standardized, objective vocabulary to advance both the science and public acceptance of the potential benefits of psychedelic medicines. We offer three reasons why the formulation of a new lexicon for psychedelic therapy is needed.

First, it is critical to clearly distinguish the therapeutic applications of psychedelic compounds from spiritual or recreational uses. To illustrate this point, we describe how terms such as “trip” may confuse the public's perception of these very different uses. Second, we discuss how a standardized nomenclature for the psychedelic substances themselves and their associated therapies can better help position psychedelics as mainstream therapeutics. Finally, a harmonized medical lexicon will allow patients to more precisely communicate with researchers and therapists about their experiences and outcomes.

We recognize that there will be practitioners and organizations that use psychedelic modalities that are rooted in spiritual, cultural, or other long-held practices and values. This paper is not a call to change religious naming conventions. We remain agnostic about the terminology used for spiritual practice or recreational activities.

We conclude by recommending ways to begin this shift in language and thinking. The recommendations we offer are aimed at therapists and organizations that will be integrated into the mainstream medical enterprise, as evidenced by, for example, their use of diagnostic codes, reimbursement by third-party payers, and participation in ongoing research.

## Shifts in the Language of Addiction Medicine

Important and ongoing changes in the language of addiction medicine serve as a model for addressing terminological confusion and stigma in psychedelic therapy. Addiction researchers have documented the negative consequences that inaccurate and pejorative language has on treatment access and efficacy (6, 9, 10). We know, for example, that stigmatizing language is internalized by individuals (self-stigma) and adversely affects self-esteem and self-efficacy, leading to worsening symptoms of depression and anxiety (11–13). Stigma inhibits access to care by preventing people from seeking treatment because they are less likely to talk about their addiction (14, 15). Importantly, stigmatizing attitudes held by health professionals may prevent patients from receiving comprehensive, quality treatment (16, 17).

The Journal of Addiction Medicine editorial board developed recommendations for revised terminology that encourage clinicians to use person-first language that focuses on the medical aspects of diseases and disorders and avoids perpetuating negative stereotypes (6). For example, Pfund et al. recommend that clinicians should avoid using evaluative terms that include any implicit assumptions about patients (10). Instead of using the term “detoxification,” for example, Saitz et al. recommend “withdrawal management” (6).

The semantic shift from “addiction” to “substance use disorder” offers another example of how words shape public perception of medical conditions and interventions. “Substance use disorder,” introduced officially in the DSM-5, marked a shift toward more objective terminology and away from value-laden concepts because terms like “abuser” and “addict” convey moral judgment and are stigmatizing.

Objective terms are not only more precise than evaluative terms, but they also minimize the transmittal of normative judgments that might interfere with patient care. Refraining from using subjective terms will ensure medical records and patient notes will be less likely to include misinterpretations or personal biases. The lexical revisions in addiction medicine may offer similar ways forward for psychedelic medicine.

For example, practitioners should avoid terms like “molly,” “shrooms,” or “mushrooms” which are hallmarks of underground or recreational use, and instead use labels based upon the substance's chemical structure or mechanism of action. These names should not be derived from the side effects of the drugs (i.e., hallucinogens). Relying upon a side effect as the basis of naming the substance is misleading and potentially stigmatizing. Table 1 contrasts stigmatizing language or slang with suggestions for more appropriate scientific terminology.

**Table 1:** Recommendations for clinically accurate terminology.

**Stigmatizing language or slang**	**Scientifically accurate terminology**
Guide, trip sitter, co-pilot, voyager, psychonaut, shaman, curandero	Psychiatrist, therapist
Psychedelics, hallucinogens	5HT_2A_ receptor agonists
- Acid, L, tabs, blotter, doses, trips	- Lysergamides: d-lysergic acid diethylamide (LSD)
- Ecstasy, E, X, XTC, rolls, beans, adam, molly	- Phenylethamines: 3,4 methylenedioxymethamphetamine (MDMA)
- Mushrooms, shrooms, shroomies, mushies, magic mushrooms, sacred mushrooms, Psilocybes, Liberty Caps, Cubes	- Tryptamines: Psilocybin, psilocin
Dropping acid, flying, frying, shrooming, rolling, X-ing, thizzing, wigging, tripping, dosing, microdosing	Taking or being prescribed a drug or medicine
Trip, journey, higher state(s) of consciousness	Therapeutic state(s) of altered consciousness
Bad trip, freaking out	Challenging experience, acute adverse psychological reaction

*Adapted from Saitz et al. (6); slang/street terminology from erowid.org (18)*.

## A Language to Advance the Field

For psychedelic medicine to become broadly accepted, the field will need to clearly circumscribe itself according to the medical model. Research organizations such as MAPS and Compass Pathways have recognized that the FDA-approval process is the ideal route for broad acceptance and access to these medicines. Therefore, a standardized scientific lexicon is necessary to differentiate psychedelic medicine from recreational, spiritual, and religious uses.

The term “trip” has historically referred to the psychedelic experience. “Tripping” conjures images of the hippie counterculture, a social movement that railed against conservative mores and challenged the political status quo. LSD was criminalized by its classification as a Schedule I substance via the Controlled Substance Act of 1967, leading to its increased presence in politics and parties, and disappearance from medical research (19). Public fear of LSD's usage was fuelled by specious claims by government officials who claimed the danger of LSD was similar to narcotics and that LSD was more dangerous than the Vietnam War (4). The media further propagated moral panic during the sixties by spreading misinformation that LSD would turn young people into hippies (4).

We find the word “trip” in contemporary biomedical contexts. A new research unit at the Pacific Neuroscience Institute is named Treatment and Research in Psychedelics (TRIP), and researchers are using the expression “bad trip” as a medical term within published research (20, 21). Media outlets continue to use the term in documentaries (e.g., NYU's “Magic Mushrooms and the Healing Trip”), and news articles (e.g., Pollan in NYT's “How Does a Writer Put a Drug Trip into Words?”) (22, 23). A recent New York Times article used “trip” six times while describing psychedelic usage in research trials (e.g., “The Next Big Addiction Treatment”) (24).

Similarly, we see the term used by organizations touting the mental health benefits of psychedelics. For example, companies such as Field Trip Health Inc. use the term “trip” in a way that conveys aspects of underground culture. This may contribute to the misconception that mental healthcare is categorically different from healthcare more generally, and that mental disorders involve moral or spiritual deficiencies that need to be addressed for healing. Adding a spiritual facet to messaging around psychiatric recovery blurs boundaries between medical use and personal growth. The term “trip” still carries negative associations with insanity, “freaking out,” “acting crazy,” or “acting funny” often perpetuated by inaccurate media portrayals of the dangers of these compounds (25, 26). No terms are purely value-neutral, but the meaning of terms like “trip” and “tripping” may be so steeped in negative assumptions that their use undermines the clinical encounter.

## Naming the Substances

Psychedelic researchers should model the naming conventions for psychedelics in ways similar to other pharmaceuticals. Antibiotics, which are named according to their structure and mechanism of action, provide one potential example. Following an existent structural model for naming avoids the misinformation, ambiguity, or confusion caused by inconsistent naming schemata.

Psychoactive agents are already categorized by structure (tryptamines, phenethylamines, and lysergamides), as well as share a mechanism of action (5HT_2A_ receptor agonist). This categorization could be used to provide psychoactive agents with labels that can be consistently applied. The clinical outcome naming system (i.e., antidepressants) should be avoided so laypersons do not mistakenly associate psychoactive substances with other drugs and their well-known side effects.

Another aspect of dividing medical and recreational uses of psychedelics is ensuring that marketing and visual representations of psychedelics support responsible use. Much like the preservation of “trip,” psychedelic therapy constituents have opted to preserve popular, common imagery that is reflective of familiarity to advertise their research or practice. For example, the use of psychedelic artwork, kaleidoscopic patterns, and surrealist images and logos may unintentionally engender or reinforce negative bias. To ensure broad access, psychedelic pharmaceutical companies should aim to provide objective language and images on public-facing material that convey scientific rigor and integrity, and do not dilute or mislead the purpose of its practice. Policymakers should begin now to develop regulations–both for psychedelics for medical uses and recreational uses–to prevent sensationalist marketing and packaging (27).

Practitioners should be familiar with terms that belong to categories of appropriate medical use, and what sets these terms apart from recreational and/or spiritual use (Table 1).

## Therapy Categorization

Psychedelic medicines appear to be catalysts or facilitating agents for psychotherapy. This is important because the use of psychedelic medicine should be understood as a necessary but not sufficient component of the full therapeutic modality that includes intensive psychotherapy. Psychedelic medicines enable the patient to access a non-normal state of consciousness, that frees them from their habitual neurobehavioral states and habits (28). They are better able to grapple with their memories, address their traumas, and actively participate in the work that healing requires. By emphasizing the healing potential of expanded states of consciousness we may avoid the stigmas associated with recreational usage.

The exploratory state of mind that is activated by psychedelics may be referred to as the “therapeutic state of altered consciousness” or “therapeutic experience.” For example, Stace and Pahnke both refer to the psychedelic experience of mystical consciousness as the place where “subject-object dichotomy (is) transcended and the empirical ego extinguished” (29, 30). More recently, Wolff et al. propose a mechanism called “psychedelic-induced belief relaxation” that can help better facilitate cognitive behavioral therapy (31). The proposed “therapeutic state of altered consciousness” or “therapeutic experience” can thus serve as an umbrella term that encapsulates the explanations for why therapies work so well in this state of mind. The “therapeutic state of altered consciousness” is also in line with Bayne and Carter's recommendation to avoid labeling psychedelic experiences as accessing “higher” states of consciousness. They illustrate that “higher” is not at all objective, citing multiple evidence-based experiments and surveys (32).

Preparation before, and integration after, the psychedelic experience are essential components of therapy (31). The preparatory phase is crucial for the patient to establish and apply intention: the patient must first understand they are an active agent of their own care, and that the therapeutic session doesn't begin and end with merely taking the psychedelic. Integrative work has already demonstrated its efficacy, and some physicians have reported that they believe it to be the most important aspect of therapy (33).

Thus, the term “assisted” used in the context of “Psychedelic Assisted Therapy” is a misnomer. Psychedelic medicines do not assist, but rather are integral in the therapeutic modality (A similar issue exists in addiction medicine, where the modifier “medically assisted” is inappropriately used to describe the use of highly effective medications to treat substance use disorder). Accurate language usage can guide physicians and patients to better accept the medicine and therapy as a combined therapeutic modality, and should do so in a way that emphasizes the process (therapy) over the product (psychedelic substance; medicine).

## The Therapeutic Relationship and Experience

Psychiatry can use language as an additional tool to shape and clarify patients' psychedelic experiences (34). Language can help frame and guide patients' experiences and ways to communicate these experiences. Physicians have a responsibility to keep their patients well-informed about psychedelics—how they work, and how they work in conjunction with the therapy being offered, how they are experienced, etc.—and need to do so in understandable terms.

Psychedelic therapists should emphasize how psychedelic experiences can be integrated into patients' lives and continued therapeutic process. Narrative medicine provides one apt arena for useful integration. Like psychedelic therapy, narrative medicine is a practice emerging into greater recognition and application. As a healthcare discipline, it prioritizes the building of relationships through a design of attentive listening that grapples with the complexities and unique qualities of individual patient circumstance (35).

Narrative medicine may be usefully incorporated into this stage of psychedelic therapy. In contrast to psychometric instruments administered by a therapist, narrative medicine prioritizes patients' own stories in framing and understanding their clinical experiences. Employing narrative medicine in psychedelic therapy can add rich qualitative data to inform both ethical and clinical perspectives.

## Recommendations

As psychedelic research expands, investigators should review the language used in consent conversations and documents, marketing materials, and publications, taking care to avoid an overreliance on metaphoric language to describe their treatments. Metaphors used to explain psychedelics should not represent anachronistic views about recreational uses.

We are not suggesting that psychedelic researchers and therapists employ a rigid lexicon, but that careful consideration be given to important aspects of clinical language, which may shape patient expectations and, more broadly, the identity of the field. The terms listed in Table 1 are suggestions for consideration and use by experienced clinicians. Psychedelic therapy instructors and educational program directors should transition their curricula toward non-stigmatizing language. It is also important to use accurate language to educate medical students, residents, and general practitioners about psychedelic therapy. In order to create consistent messaging and to help shape positive views of this emerging field, it would be best for the medical population (doctors, students, etc.) to adopt this language now. A statement from the American Psychiatric Association (APA) encouraging the use of non-stigmatizing and precise language would be instrumental in achieving this directive.

Finally, and critically important, a harmonized medical lexicon will facilitate coding and billing to third-party payers.

## Conclusion

Psychedelic medicines hold great potential to become mainstream therapeutics. Scientifically accurate terminology will foster the development of the field, broadening acceptance of and widening access to psychedelic medicines. While it is important to note that there will be groups who remain committed to cultural and religious understandings of psychedelic compounds, our recommendations are directed specifically to health care professionals who provide treatment according to the medical model. A harmonized medical lexicon can help assuage concerns about psychedelics that remain associated with stigmatizing and anachronistic language.

## Data Availability Statement

The original contributions presented in the study are included in the article/supplementary material, further inquiries can be directed to the corresponding author/s.

## Author Contributions

AB and DS contributed to the conception and design of the perspective article. AB wrote the first draft of the manuscript. All authors contributed to manuscript revision, read, and approved the submitted version.

## Conflict of Interest

DS consults COMPASS (for pay), and MAPS (pro bono). The remaining author declares that the research was conducted in the absence of any commercial or financial relationships that could be construed as a potential conflict of interest.

## Publisher's Note

All claims expressed in this article are solely those of the authors and do not necessarily represent those of their affiliated organizations, or those of the publisher, the editors and the reviewers. Any product that may be evaluated in this article, or claim that may be made by its manufacturer, is not guaranteed or endorsed by the publisher.
